# Remote Assessment of Platelet Function in Patients with Acute Stroke or Transient Ischaemic Attack

**DOI:** 10.1155/2017/7365684

**Published:** 2017-05-24

**Authors:** Philip M. Bath, Jane May, Katie Flaherty, Lisa J. Woodhouse, Natalia Dovlatova, Sue C. Fox, Timothy J. England, Kailash Krishnan, Thompson G. Robinson, Nikola Sprigg, Stan Heptinstall, TARDIS Investigators

**Affiliations:** ^1^Stroke Trials Unit, Division of Clinical Neuroscience, University of Nottingham, City Hospital Campus, Nottingham NG5 1PB, UK; ^2^Stroke, Nottingham University Hospitals NHS Trust, City Hospital Campus, Nottingham NG5 1PB, UK; ^3^Platelet Solutions Ltd., Division of Clinical Neuroscience, University of Nottingham, Queen's Medical Centre Campus, Nottingham NG7 2UH, UK; ^4^Platelet Research Group/Stroke, Division of Clinical Neuroscience, University of Nottingham, Queen's Medical Centre Campus, Nottingham NG7 2UH, UK; ^5^Vascular Medicine, Division of Medical Sciences and GEM, School of Medicine, University of Nottingham, Nottingham, UK; ^6^Department of Cardiovascular Sciences, University of Leicester, Glenfield Hospital, Leicester LE3 9QP, UK; ^7^University of Nottingham, Nottingham, UK

## Abstract

**Background:**

The TARDIS trial assessed the safety and efficacy of intensive versus guideline antiplatelet agents given for one month in patients with acute stroke or TIA. The aim of this substudy was to assess the effect of antiplatelet agents taken at baseline on platelet function reactivity and activation.

**Methods:**

Platelet function, assessed by remotely measured surface expression of P-selectin, was assessed in patients at their time of randomisation. Data are median fluorescence values.

**Results:**

The aspirin P-selectin test demonstrated that platelet expression was lower in 494 patients taking aspirin than in 162 patients not: mean 210 (SD 188) versus 570 (435), difference 360.3 (95% CI 312.2–408.4) (2*p* < 0.001). Aspirin did not suppress P-selectin levels below 500 units in 23 (4.7%) patients. The clopidogrel test showed that platelet reactivity was lower in 97 patients taking clopidogrel than in 585 patients not: 655 (296) versus 969 (315), difference 314.5 (95% CI 247.3–381.7) (2*p* < 0.001). Clopidogrel did not suppress P-selectin level below 860 units in 24 (24.7%) patients.

**Conclusions:**

Aspirin and clopidogrel suppress stimulated platelet P-selectin, although one-quarter of patients on clopidogrel have high on-treatment platelet reactivity. Platelet function testing may be performed remotely in the context of a large multicentre trial. Trial registration ISRCTN47823388.

## 1. Introduction

Secondary prevention after ischaemic stroke (IS) or transient ischaemic attack (TIA) comprises blood pressure and lipid lowering and antithrombotic therapy, either anticoagulation for atrial fibrillation (in 20% of patients) or antiplatelet therapy for noncardioembolic stroke/TIA (in 80%) [1–6]. Antiplatelets are effective at reducing recurrent events after IS or TIA, as shown in multiple large trials and meta-analyses [7–13], and UK guidelines recommend the following antiplatelet regimes (in order): clopidogrel, combined aspirin and dipyridamole, aspirin alone, or dipyridamole alone [14, 15]. In contrast to the use of blood pressure and lipid lowering drugs and anticoagulants, where biomarkers (blood pressure, blood lipids, and coagulation screen) can be used to adjust treatment, antiplatelet drugs are given in a “fire and forget” manner since there are no validated, widely available, or inexpensive tests of platelet function that measure platelet activity reliably and reproducibly, correlate with recurrent events, and can be measured remotely from a dedicated platelet laboratory. As a result, antiplatelet therapy is given routinely without knowledge of whether it is working in a particular patient.

Unfortunately, a significant minority of patients (≥35%) do not respond adequately to clopidogrel [16–25]; and a similar but lesser problem exists for aspirin [16, 18, 20, 26, 27]. A number of laboratory-based systems for measuring platelet function exist, including aggregation and commercial systems such as Multiplate and VerifyNow [27–29]. However, these approaches cannot be used remotely from the patient (i.e., by general practitioners or at hospitals without specialist equipment). Some of the point-of-care assays (PFA-100 and VerifyNow) have shown very poor agreement [29, 30].

Surface platelet expression of P-selectin (CD62P, PADGEM, Platelet Solutions Ltd.) may be measured remotely in fixed blood [31] and correlates with other measures of platelet function testing [32]. Using this approach, a small study in 100 patients with acute coronary syndromes (ACS) found that high on-treatment platelet reactivity with clopidogrel was present in 42% of patients and that these had an increased risk of subsequent cardiovascular death, acute coronary syndrome, or stent thrombosis [33]. The present study extends this work into patients with ischaemic stroke or TIA and assessed ex vivo responsiveness to aspirin and clopidogrel at baseline in patients recruited into the ongoing TARDIS antiplatelet trial. The specific aim of the substudy was to assess the effect of antiplatelet agents taken at baseline on platelet reactivity assessed using measurements of platelet surface P-selectin expression. We hypothesised that whilst antiplatelet agents would suppress platelet surface P-selectin expression in most patients, some would not exhibit such suppression, especially when taking clopidogrel.

## 2. Methods

### 2.1. TARDIS

TARDIS assessed the safety and efficacy of intensive versus guideline based antiplatelet therapy in patients with acute ischaemic stroke or TIA [34, 35]. In brief, TARDIS was an international parallel-group prospective randomised open-label blinded-endpoint controlled trial (trial registration ISRCTN47823388). Patients with acute ischaemic stroke or TIA were randomised within 48 hours of ictus to one month of open-label intensive antiplatelet therapy—combined aspirin (300 mg load and then 75 mg daily), clopidogrel (300 mg load and then 75 mg daily), and dipyridamole (typically modified release 200 mg twice daily)—versus guideline antiplatelet therapy—combined aspirin and dipyridamole, or clopidogrel alone (dosing as above). Randomisation was made irrespective of what antiplatelet therapy, if any, patients were taking prior to enrolment. The primary outcome was stroke recurrence and its severity at 90 days [34, 35]. Gastroprotection (with either a protocol pump inhibitor or H2 antagonist) was given according to local guidelines with the proviso that omeprazole and esomeprazole should be avoided since they may attenuate the effects of clopidogrel. The trial was coordinated by a Trial Management Committee, overseen by a Trial Steering Committee and International Advisory Committee, and safety was monitored by an Independent Data and Safety Monitoring Committee [34, 35].

The aim of this substudy was to assess the ex vivo effect of antiplatelet agents taken at baseline on platelet activation. Patients were grouped by antiplatelet agent(s) taken prior to randomisation (as declared during collection of baseline information), either regularly prior to stroke/TIA or given in hospital after presentation and prior to enrolment into TARDIS, that is, within 48 hours of their index event. Information on antiplatelet agents was obtained through history taking at the time of collecting baseline data and not by review of prescription charts or general practice records. Antiplatelet groups comprised aspirin alone, aspirin and clopidogrel (a trial protocol violation [34]), aspirin and dipyridamole, clopidogrel alone, and no antiplatelet prior to randomisation. Groups were also combined into aspirin versus no aspirin, and clopidogrel versus no clopidogrel. Duration and dose of antiplatelet drugs are not presented in view of the unreliable nature of this information when taken by history at baseline.

### 2.2. P-Selectin Assay

A single blood biomarker assessing platelet function—platelet surface P-selectin expression—was used with measurements at baseline (i.e., prior to the receipt of the randomised antiplatelet regimen) and at day 7 after randomisation. P-selectin is derived from alpha-granules in resting platelets and becomes exposed on the cell surface membrane upon platelet activation and granule release. The level of P-selectin inside platelets is independent of that produced by endothelial cells; this contrasts with measurement of circulating soluble (plasma) P-selectin that depends on both cell types and as reported previously in stroke [36]. Platelet P-selectin expression was chosen since blood can be taken and processed at multiple clinical sites with measurement at a central core laboratory [31, 33]; no other technique allows such remote assessment of platelet function. Since TARDIS is still running, it was not possible to lock the database and analyse on-treatment P-selectin measurements; as a result, the following results relate only to analyses using baseline prerandomisation P-selectin data.

Blood was taken by research staff who were trained in venepuncture and sample preparation; samples were taken immediately after randomisation in the Acute Stroke Unit. Platelet surface P-selectin expression was measured using commercial kits (Platelet Solutions Ltd. [PSL], Nottingham, UK). Citrate anticoagulated blood was collected and kept at 37°C (by a dry heat pad kept in an insulation pouch) prior to incubation with platelet stimulants [31] comprising either adenosine diphosphate (ADP) for testing clopidogrel (i.e., clopidogrel test) or arachidonic acid (AA) for testing aspirin (i.e., aspirin test), both with added potentiating agents at low concentration and EDTA to prevent aggregation; unstimulated samples were also collected to provide information on baseline expression. After 5 minutes of platelet stimulation, fixing solution (PAMFix, PSL) was added [32]. Blood processing was performed by the bedside to reduce time from collection to stimulation. Fixed samples (which are stable for at least 9 days [31]) were posted for flow cytometric analysis in the Nottingham flow cytometry laboratory, typically within 6 days of being taken.

Platelet reactivity was assessed as platelet surface P-selectin expression as described previously [31]. In brief, samples of fixed blood were incubated with fluorescent antibodies to the platelet specific marker CD61 (used to identify platelets) and CD62P (P-selectin). FACSflow was added to samples immediately prior to analysis using a flow cytometer (Becton Dickinson FACSCanto II). Median Fluorescence (MF) of each sample (3000 platelets) was recorded as a measure of platelet surface P-selectin expression. Data were then entered into the main TARDIS database.

The platelet surface P-selectin expression assay has been compared previously with optical and whole blood aggregometry and aggregometry measurements agreed well with data obtained in vitro and ex vivo following inhibition with aspirin, cangrelor, or clopidogrel treatment [31, 37]. The technique does not assess the effects of dipyridamole, in contrast to some other approaches [38, 39].

### 2.3. Statistics

A subset of data from the TARDIS database was extracted (patients with a P-selectin measurement and without randomisation codes) to allow statistical analyses. Results are given as number (%), median [interquartile range], or mean (standard deviation, SD). “Bee swarm” plots are used to present the distribution of ADP and AA related P-selectin expression in subgroups. Cut points for ADP (500, 625, and 860 units [33]) and aspirin (400, 500 units) were chosen prospectively and used to define responsiveness (level lower than cut-off point) versus no response. The cut points were chosen either because they have been used before [33] or to provide guidance for future studies; in particular, we will test these in analysis of the relationship between randomised TARDIS treatment, P-selectin expression, and clinical endpoints. Statistical analyses were performed using SAS software (version 9.3).

## 3. Results

### 3.1. Patients

The trial started recruitment on April 7, 2009, and data were extracted from the database on November 14, 2016, following recruitment of 3096 patients from 106 hospital sites in the UK. Of these, 689 (22.3%) patients from 23 recruiting sites (21.7%) had a P-selectin expression measurement at baseline; one patient was not included in subsequent analyses having presented on triple antiplatelets (*n* = 1, a protocol violation [34]). The characteristics of the patients are shown in Table 1. The average age of patients was 68.6 years, 34.7% were women, and 75.0% were recruited with an index event of ischaemic stroke.

The majority of patients (62.4%) had received aspirin alone prior to randomisation; 16.1% patients had taken nothing, and smaller numbers had taken clopidogrel alone (7.8%), combined aspirin and dipyridamole (7.3%), or combined aspirin and clopidogrel (6.1%) (Table 1). When assessed as whether on aspirin or clopidogrel at baseline, 75.8% of patients were taking aspirin and 13.9% clopidogrel. Gastroprotection was taken in 257 (37.3%) of patients in the substudy, with most of these (237, 92.2%) receiving a proton pump inhibitor.

### 3.2. P-Selectin in Response to Arachidonic Acid (Aspirin Test)

AA-stimulated platelet surface P-selectin expression was lower in patients on aspirin than those who were not taking it, mean difference in fluorescence 360.3 (95% CI 312.2, 408.4; 2*p* < 0.001) (Table 2). Using a cut-off point of 500 MF, 23 (4.7%) patients on aspirin did not have suppressed P-selectin expression in response to AA (Table 2). Acute aspirin administration was associated with a nonsignificant tendency to a lower P-selectin expression in response to AA as compared with chronic consumption (Table 3), perhaps reflecting that acute administration of aspirin after stroke involves a loading dose (300 mg) rather than a daily maintenance dose of 75 mg.

**Figure 1:**
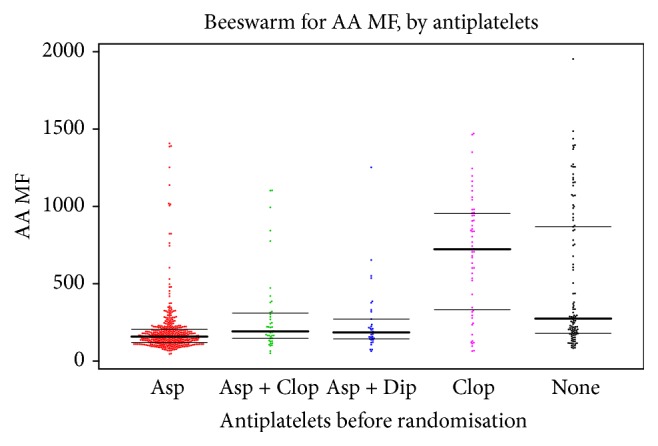
P-selectin in response to arachidonic acid (AA, aspirin test), by antiplatelet medication taken prior to randomisation. Data are median fluorescence (MF). The horizontal lines show the median and interquartile values.

AA-stimulated P-selectin expression varied between antiplatelet groups being lower in those patients taking aspirin alone, combined aspirin and dipyridamole, or combined aspirin and clopidogrel, as compared with clopidogrel alone or no antiplatelets (Table 4, Figure 1). AA-stimulated P-selectin was lower on aspirin than on combined aspirin and clopidogrel, mean difference −98.2 (95% CI −183.7, −12.8; 2*p* = 0.025) (Table 4). There was no significant difference in platelet surface P-selectin expression between patients on aspirin alone as compared with those on combined aspirin and dipyridamole, mean difference −58.3 (95% CI −140.2, 23.7; 2*p* = 0.16). Clopidogrel alone was associated with higher AA-stimulated P-selectin expression than in patients taking no antiplatelet agents (mean difference 183.6; 95% CI 50.0, 317.3; 2*p* = 0.0075).

### 3.3. P-Selectin in Response to ADP (Clopidogrel Test)

ADP-stimulated platelet surface P-selectin expression was lower in patients on clopidogrel than those who were not taking it, mean difference in fluorescence −314.5 (95% CI −381.7, −247.3) (Table 2). Using a cut-off point of 860 MF [33], 24 (24.7%) of patients taking clopidogrel did not have suppressed P-selectin expression (Table 2). P-selectin expression did not differ in patients who only received acute treatment with clopidogrel as compared with those who had taken clopidogrel prior to their stroke (Table 3).

**Figure 2:**
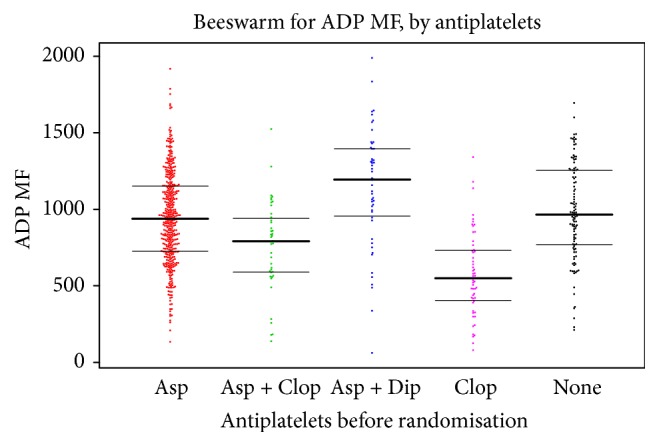
P-selectin in response to adenosine diphosphate (ADP, clopidogrel test), by antiplatelet medication taken prior to randomisation. Data are median fluorescence (MF). The horizontal lines show the median and interquartile values.

ADP-stimulated P-selectin varied between groups receiving different antiplatelet agents being lower in the group of patients taking clopidogrel versus aspirin-based therapy or no antiplatelets (Table 4, Figure 2). ADP-stimulated P-selectin was lower on clopidogrel than on combined aspirin and clopidogrel, mean difference −180.6 (95% CI −298.3, −63.0; *p* = 0.003) (Table 4). As expected, aspirin alone had no effect on ADP-stimulated P-selectin expression in comparison with patients taking no antiplatelets, mean difference −38.6 (95% CI −104.5, 27.4; 2*p* = 0.25) (Table 4).

### 3.4. P-Selectin with No Stimulation

**Figure 3:**
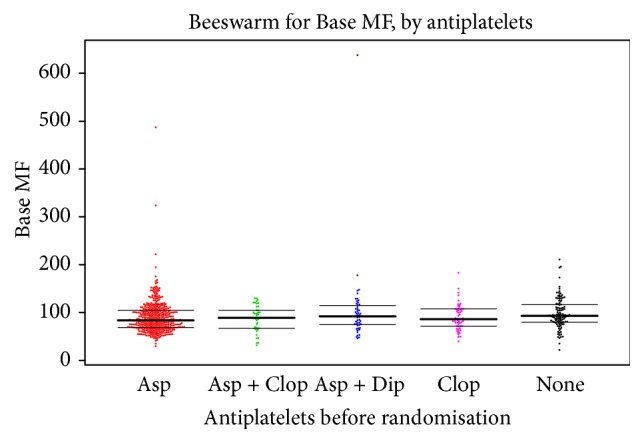
P-selectin with no stimulation, by antiplatelet medication taken prior to randomisation. Data are median fluorescence (MF). The horizontal lines show the median and interquartile values.

Platelet P-selectin expression did not vary by presence or absence of antiplatelets prior to randomisation (Table 5, Figure 3).

## 4. Discussion

Remote assessment of platelet function in patients with acute stroke or TIA is feasible and reveals that aspirin reduces AA-stimulated platelet surface P-selectin expression, and clopidogrel lowers ADP-stimulated platelet P-selectin. Using predefined cut-offs, whilst AA-stimulated P-selectin was suppressed in 95% of patients in response to aspirin, clopidogrel failed to reduce ADP-stimulated P-selectin in 24.7% of patients receiving the drug. The combination of aspirin and clopidogrel was associated with higher AA and ADP-stimulated P-selectin levels than either agent alone. Whilst acute aspirin was associated with an insignificant tendency to a lower AA-stimulated P-selectin than chronic aspirin treatment, there was no difference in ADP-stimulated P-selectin between acute and chronic clopidogrel consumption. Unstimulated samples showed low levels of P-selectin expression irrespective of whether antiplatelet agent had been taken beforehand.

Remote assessment of platelet function is unique with this assay system. Alternative approaches such as whole blood aggregation, platelet rich plasma aggregation, Multiplate, and VerifyNow require specific equipment that needs to be proximal to patients and is usually found in specialist platelet laboratories. The present system is comparable to measurement of blood lipids or an international normalised ratio: a blood sample is taken at, say, a clinic or general practice, processed using a simple kit and sent (by post in this study) to a core laboratory (not necessarily the one used here) where it is analysed using flow cytometry, and a result then returned for interpretation and conveyance to the patient. This approach was feasible across multiple sites in the UK with the core laboratory in Nottingham. These results suggest that response to aspirin and clopidogrel therapy can be monitored remotely, much as they are for statins and oral anticoagulants.

Attenuation of platelet expression of ADP- and AA-stimulated P-selectin has been demonstrated previously for clopidogrel and aspirin, respectively [31]. However, a significant minority of patients (≥35%) do not respond adequately to clopidogrel [16–25], and a similar but lesser problem exists for aspirin [16, 18, 20, 26, 27]. Measurement of ADP-stimulated P-selectin expression has been shown to identify individuals with acute coronary syndromes (ACS) who have high on-treatment platelet reactivity with clopidogrel [33]; these patients had an increased risk of subsequent cardiovascular death, acute coronary syndrome, or stent thrombosis suggesting that the P-selectin measurement has prognostic significance. Similarly, high on-treatment platelet reactivity (alternatively called resistance) in patients taking clopidogrel due to the presence of the CYP2C19 loss-of-function allele is associated with a failure for this antiplatelet to add to aspirin in preventing recurrence after minor stroke and TIA [40]. A recent review summarises current data on the magnitude of this problem [41]. Since the TARDIS trial has not completed follow-up and data validation, it is not possible currently to assess whether high on-treatment platelet reactivity on clopidogrel is prognostic for recurrent cerebrovascular events such as stroke and TIA; these data will be analysed once the TARDIS main results have been published.

A novel finding in this study was that the addition of clopidogrel to aspirin was associated with higher AA-stimulated P-selectin levels than aspirin alone, and the addition of aspirin to clopidogrel was associated with higher ADP-stimulated P-selectin levels than clopidogrel alone. A nonsignificant tendency for the same phenomenon was seen whereby higher AA-stimulated P-selectin levels were seen in the presence of aspirin and dipyridamole as compared to aspirin alone. These findings can be interpreted in three ways. First, combining antiplatelets may interfere or reduce the effect of each agent alone, a finding that has not been previously reported. Whether such an effect might reduce clinical effectiveness is unclear but this was not seen in the European Stroke Prevention Study-2 (ESPS-2) which found that combining aspirin and dipyridamole gave twice the stroke preventative effect of either antiplatelet agent alone when compared with placebo [9]. Second, differences in doses of antiplatelets might explain these findings. Last, the particular assay used here might detect apparent interference when none is actually present, that is, a false positive finding. Whatever the mechanism, the phenomenon of apparent interference between antiplatelets requires further study. The finding that acute aspirin administration is associated with potentially lower P-selectin expression than chronic administration supports the importance of loading patients with aspirin after ischaemic stroke/TIA, as done in TARDIS.

The present study involved a large number of patients with recent stroke or TIA, and they were taking a variety of antiplatelet regimes, for varying periods of time (acute versus chronic). Importantly the data come from a large high-fidelity trial with prospective data collection [34, 35]. However, the study has several caveats. First, only a minority (22.3%) of patients from the main trial were recruited into this substudy, and these differed in that they were more likely to be male and have a history of previous ischaemic stroke. Nevertheless, this should not have affected the ability of the study to detect differences in P-selectin between patients taking different antiplatelet agents. Further, the study was much larger than earlier ones using the P-selectin assay [31, 33, 37]. Second, the antiplatelet drug history came from the patient (or carer) reflecting their memory of what had been taken recently, and it was not possible to confirm this by direct measurement of drugs or metabolites. Hence, patients may not have been taking the drug(s) they said they were on, although such noncompliance reflects real-world reality. Equally, some patients reporting taking no antiplatelet drugs may have been taking aspirin; this is apparent in the proportion of patients with low P-selectin levels on the aspirin test who reported taking no antiplatelet drug and the presence of apparent cross-over effects whereby the aspirin test gave low values in patients on clopidogrel. There may be other causes of low values in addition to antiplatelet therapy, potentially including dietary sources of salicylate [42]. Third, this study assessed platelet reactivity in a cross-sectional manner and multiple assessments, as in longitudinal studies [39], might be superior. Fourth, in the case of aspirin administration, a mixed group of patients on acute and chronic antiplatelet therapy were assessed, and potential differences in antiplatelet effect were seen; again, this reflects real-world reality. Fourth, there is the possibility that low levels of platelet reactivity could be due to prior platelet activation in the setting of an acute event and reflect possible platelet exhaustion, a possibility that would affect measurements performed using a wide range of approaches to measurement of platelet function, including platelet surface P-selectin expression. Finally, patients would ideally have been rested prior to venepuncture to avoid activity-related changes in platelet function; unfortunately, it was not possible to mandate this in the acute stroke unit/busy TIA clinic environment.

In summary, it is feasible to assess inhibition of platelet function by quantifying P-selectin expression with the assay kit used here in a central laboratory, remote from the site of clinical assessment and venepuncture. P-selectin measures are determined by antiplatelet therapy although a sizable minority of patients on clopidogrel appear to be insensitive to therapy. Assessment of the relationship between P-selectin levels and subsequent clinical outcome will be assessed in a further publication once the TARDIS trial was completed and the data base locked, and data can be analysed taking account of treatment assignment.

## Figures and Tables

**Table 1:** Characteristics of patients at baseline/prerandomisation who have P-selectin data and were on antiplatelet therapy either taken before stroke or given acutely after stroke. Data are number (%), median [interquartile range], or mean (standard deviation). Comparison across all antiplatelet groups by Chi-square test, Kruskal-Wallis test, or one-way analysis of variance.

	Whole trial	With P-selectin	Aspirin	Asp/Dip	Asp/Clop^†^	Clopidogrel	None	2*p*
Number	3096	689	430	50	42	54	111	
Age (years)^‡^	69.0 (10.1)	68.6 (10.4)	68.9 (10.3)	70.3 (10.7)	70.5 (9.7)	68.9 (11.4)	66.3 (9.9)	0.078
Sex, male (%)^‡^	1945 (62.8)	450 (65.3)	277 (64.4)	33 (66.0)	26 (61.9)	35 (64.8)	78 (70.3)	0.81
History (%)								
Hypertension	1827 (59.0)	400 (58.1)	276 (64.2)	27 (54.0)	28 (66.7)	28 (51.9)	41 (36.9)	<0.001
Hyperlipidaemia	1317 (42.5)	278 (40.3)	181 (42.1)	24 (48.0)	20 (47.6)	21 (38.9)	31 (27.9)	0.24
Diabetes	590 (19.1)	144 (20.9)	101 (23.5)	6 (12.0)	13 (31.0)	8 (14.8)	16 (14.4)	0.03
Previous stroke	349 (11.3)	67 (9.7)	32 (7.4)	15 (30.0)	10 (23.8)	7 (13.0)	3 (2.7)	<0.001
Previous TIA	337 (10.9)	68 (9.9)	36 (8.4)	17 (34.0)	4 (9.5)	10 (18.5)	1 (0.9)	<0.001
Ischaemic heart disease	403 (13.0)	71 (10.3)	51 (11.9)	7 (14.0)	5 (11.9)	4 (7.4)	3 (2.7)	0.05
Peripheral vascular disease	70 (2.3)	8 (1.2)	4 (0.9)	2 (4.0)	1 (2.4)	0 (0.0)	1 (0.9)	0.46
Smoking								
Current (%)	784 (25.7)	172 (25.4)	111 (26.1)	9 (18.4)	10 (23.8)	9 (17.0)	32 (30.2)	0.32
Past (%)	1160 (38.0)	244 (36.0)	162 (38.0)	22 (44.9)	11 (26.2)	15 (28.3)	33 (31.1)	0.16
Never (%)	1108 (36.3)	262 (38.6)	153 (35.9)	18 (36.7)	21 (50.0)	29 (54.7)	41 (38.7)	0.051
Alcohol								
High > 21 upw (%)	291 (9.7)	82 (12.3)	50 (11.9)	8 (17.0)	4 (10.0)	6 (11.8)	14 (13.5)	0.85
Moderate 1–21 upw (%)	1696 (56.5)	385 (57.9)	242 (57.5)	24 (51.1)	23 (57.5)	32 (62.7)	62 (59.6)	0.82
None (%)	1014 (33.8)	198 (29.8)	129 (30.6)	15 (31.9)	13 (32.5)	13 (25.5)	28 (26.9)	0.87
Index event (%)								
Ischaemic stroke	2143 (69.2)	517 (75.0)	315 (73.3)	36 (72.0)	32 (76.2)	43 (79.6)	90 (81.1)	0.44
TIA	953 (30.8)	172 (25.0)	115 (26.7)	14 (28.0)	10 (23.8)	11 (20.4)	21 (18.9)	0.44
Stroke								
NIHSS (/42)^‡^	3.0 [2.0, 5.0]	3.0 [2.0, 5.0]	3.0 [2.0, 5.0]	3.0 [1.5, 4.0]	3.0 [2.0, 4.5]	3.0 [2.0, 5.0]	3.0 [1.0, 5.0]	0.95
Alteplase (%)^‡^	336 (15.7)	94 (18.2)	44 (14.0)	6 (16.7)	6 (18.8)	11 (25.6)	27 (30.0)	0.0078
TIA								
ABCD2 (/7)^‡^	5.0 [5.0, 6.0]	5.0 [5.0, 6.0]	5.0 [5.0, 6.0]	5.5 [5.0, 6.0]	6.0 [5.0, 7.0]	5.0 [5.0, 6.0]	6.0 [5.0, 6.0]	0.18
≥TIA in previous week (%)	183 (19.2)	27 (15.7)	15 (13.0)	4 (28.6)	4 (40.0)	2 (18.2)	2 (9.5)	0.11
TOAST (%)								
Cardioembolic	133 (4.3)	29 (4.3)	20 (4.7)	0 (0.0)	3 (7.3)	1 (1.9)	5 (4.5)	0.39
Large artery	1225 (40.1)	256 (37.6)	178 (42.0)	13 (26.0)	10 (24.4)	16 (30.2)	39 (35.5)	0.029
Lacunar	490 (16.0)	130 (19.1)	68 (16.0)	15 (30.0)	9 (22.0)	16 (30.2)	21 (19.1)	0.028
Mixed	22 (0.7)	4 (0.6)	2 (0.5)	1 (2.0)	0 (0.0)	0 (0.0)	1 (0.9)	0.64
Unknown	1181 (38.6)	261 (38.4)	156 (36.8)	21 (42.0)	19 (46.3)	20 (37.7)	44 (40.0)	0.74
Systolic BP (mmHg)^‡^	143.5 (18.2)	141.8 (18.2)	142.3 (18.6)	138.4 (18.0)	139.8 (18.5)	143.7 (16.6)	141.8 (17.0)	0.52
Onset to randomisation [hrs]^‡^	29.3 [21.8,39.6]	29.8 [22.3,40.3]	29.7 [22.5,40.1]	30.5 [21.6,43.3]	32.5 [24.8,43.4]	30.0 [23.5,41.2]	29.2 [17.5,39.0]	0.23
Drug history (%)								
In hospital								
Aspirin *f*	2197 (71.0)	463 (67.2)	398 (92.6)	40 (80.0)	25 (59.5)	0 (0.0)	0 (0.0)	<0.001
Clopidogrel *f*	208 (6.7)	78 (11.3)	0 (0.0)	0 (0.0)	30 (71.4)	47 (87.0)	0 (0.0)	<0.001
Dipyridamole *f*	154 (5.0)	36 (5.2)	0 (0.0)	35 (70.0)	0 (0.0)	0 (0.0)	0 (0.0)	<0.001
Antihypertensives	1742 (56.3)	383 (55.6)	262 (60.9)	28 (56.0)	29 (69.0)	26 (48.1)	37 (33.3)	<0.001
Lipid lowering	1387 (44.8)	301 (43.7)	196 (45.6)	22 (44.0)	28 (66.7)	25 (46.3)	29 (26.1)	<0.001
Diabetes medication	485 (15.7)	116 (16.8)	85 (19.8)	6 (12.0)	11 (26.2)	6 (11.1)	8 (7.2)	0.0054
Gastroprotection medication	1343 (43.4)	257 (37.3)	165 (38.4)	19 (38.0)	15 (35.7)	22 (40.7)	36 (32.4)	0.80

One patient on triple antiplatelet therapy and one patient on dipyridamole alone (protocol violations) are excluded.

^†^Protocol violation [34]; ^‡^Minimisation factor used during randomisation; *f* number (%) of those given the drug following stroke/TIA and prior to randomisation. Asp: aspirin; BP: blood pressure; Clop: clopidogrel; Dip: dipyridamole; PACS: partial anterior circulation syndrome; TACS: total anterior circulation syndrome.

**Table 2:** P-selectin (median fluorescence) in response to arachidonic acid (AA, aspirin test) and adenosine diphosphate (ADP, clopidogrel test) by antiplatelet medication taken prior to randomisation, for aspirin versus no aspirin and clopidogrel versus no clopidogrel. Data are number (%) or mean (standard deviation, SD) with 95% confidence intervals. Comparisons by Chi-square test or one-way analysis of variance.

	No aspirin	Aspirin	Difference	2*p*	No clopidogrel	Clopidogrel	Difference	2*p*
*AA*								
Number (%)	162 (24.1)	494 (20.4)			559 (20.2)	97 (29.5)		
Mean (SD)	570 (435)	210 (188)	360.3 (312.2, 408.4)	<0.001	261 (280)	515 (389)	−254.1 (−318.6, − 189.6)	<0.001
<400 (%)	83 (51.2)	463 (93.7)	−42.5 (−50.5, − 34.5)	<0.001	495 (88.6)	51 (52.6)	36 (25.7, 46.3)	<0.001
<500 (%)	86 (53.1)	471 (95.3)	−42.3 (−50.2, − 34.4)	<0.001	503 (90)	54 (55.7)	34.3 (24.1, 44.5)	<0.001

*ADP*								
Number (%)	165 (24.5)	517 (21.3)			585 (21.1)	97 (29.5)		
Mean (SD)	854 (361)	947 (318)	−93.4 (−151.1, − 35.7)	0.0015	969 (315)	655 (296)	314.5 (247.3,381.7)	<0.001
<860 (%) [33]	82 (49.7)	211 (40.8)	8.9 (0.2, 17.6)	0.045	220 (37.6)	73 (75.3)	−37.7 (−47.1, −28.2)	<0.001
<625 (%)	48 (29.1)	77 (14.9)	14.2 (6.6, 21.8)	<0.001	75 (12.8)	50 (51.5)	−38.7 (−49, −28.4)	<0.001
<500 (%)	30 (18.2)	38 (7.4)	10.8 (4.5, 17.1)	<0.001	38 (6.5)	30 (30.9)	−24.4 (−33.8, −15)	<0.001

**Table 3:** P-selectin (median fluorescence) in response to arachidonic acid (AA) and adenosine diphosphate (ADP) by antiplatelet medication taken prior to randomisation split by whether treatment was only given acutely after stroke/TIA but prior to randomisation (i.e., none before stroke) or had been taken chronically prior to stroke/TIA (whether or not also taken after stroke and before randomisation). Data are number (%) or mean (standard deviation). Comparisons by Chi-square test or one-way analysis of variance. Highlighted data reflects comparisons that are significant statistically.

	Aspirin		2*p*	Asp/Clop^†^		2*p*	Asp/Dip		2*p*	Clopidogrel		2*p*
	Acute	Chronic		Acute	Chronic		Acute	Chronic		Acute	Chronic	
*AA*												
Number (%)	270 (61.5)	169 (38.5)		11 (84.6)	2 (15.4)		15 (50)	15 (50)		36 (54.5)	30 (45.5)	
Mean (SD)	190 (155)	222 (203)	0.065	193 (91)	272 (42)	0.27	308 (307)	197 (125)	0.21	**733 (398)**	**522 (372)**	**0.03**
<400 (%)	260 (96.3)	156 (92.3)	0.068	10 (90.9)	2 (100)	0.66	12 (80)	14 (93.3)	0.28	**9 (25)**	**15 (50)**	**0.036**
<500 (%)	263 (97.4)	160 (94.7)	0.14	11 (100)	2 (100)	1.00	12 (80)	14 (93.3)	0.28	10 (27.8)	15 (50)	0.064

*ADP*												
Number (%)	271 (60.2)	179 (39.8)		11 (84.6)	2 (15.4)		21 (50)	21 (50.0)		36 (54.5)	30 (45.5)	
Mean (SD)	928 (282)	967 (328)	0.18	757 (262)	1010 (57)	0.22	**1276 (350)**	**938 (428)**	**0.008**	616 (267)	578 (304)	0.60
<860 (%) [33]	116 (42.8)	66 (36.9)	0.21	7 (63.6)	0 (0)	0.097	**3 (14.3)**	**9 (42.9)**	**0.040**	30 (83.3)	25 (83.3)	1.00
<625 (%)	35 (12.9)	26 (14.5)	0.63	4 (36.4)	0 (0)	0.31	**1 (4.8)**	**6 (28.6)**	**0.038**	21 (58.3)	18 (60.0)	0.89
<500 (%)	19 (7)	11 (6.1)	0.72	1 (9.1)	0 (0)	0.66	1 (4.8)	3 (14.3)	0.29	14 (38.9)	12 (40.0)	0.93

^†^Protocol violation [34].

**Table 4:** P-selectin (median fluorescence) in response to ADP and arachidonic acid (AA), by antiplatelet medication taken prior to randomisation, whether before or after stroke/TIA. Data are number (%) or mean (standard deviation). Comparisons by Chi-square test or one-way analysis of variance.

	All	Aspirin	Asp/Dip	Asp/Clop^†^	Clopidogrel	None	2*p*
*AA*							
Number (%)	656 (21.2)	418 (20.5)	42 (25.6)	33 (15.3)	54 (34.2)	108 (21.1)	
Mean (SD)	299 (312)	198 (172)	296 (269)	256 (227)	693 (381)	509 (448)	<0.001
2*p*, none versus	—	<0.001	0.002	0.005	<0.001	—	—
<400 (%)	546 (83.2)	398 (95.2)	35 (83.3)	29 (87.9)	15 (27.8)	68 (63)	<0.001
<500 (%)	557 (84.9)	404 (96.7)	37 (88.1)	29 (87.9)	16 (29.6)	70 (64.8)	<0.001

*ADP*							
Number (%)	682 (22)	424 (20.8)	42 (25.6)	50 (23.3)	54 (34.2)	110 (21.4)	
Mean (SD)	925 (331)	944 (298)	758 (296)	1139 (388)	578 (276)	983 (316)	<0.001
2*p*, none versus	—	0.54	0.10	<0.001	<0.001	—	—
<860 (%) [33]	293 (43)	172 (40.6)	27 (64.3)	11 (22)	45 (83.3)	37 (33.6)	<0.001
<625 (%)	125 (18.3)	55 (13)	15 (35.7)	6 (12)	34 (63)	14 (12.7)	<0.001
<500 (%)	68 (10)	28 (6.6)	6 (14.3)	3 (6)	23 (42.6)	7 (6.4)	<0.001

One patient on triple antiplatelet therapy (ADP 487 IU, AA 161 IU) and one patient on dipyridamole alone (ADP 1562 IU) (protocol violations) are excluded.

^†^Protocol violation [34].

**Table 5:** P-selectin (median fluorescence) with no stimulation, by antiplatelet medication taken prior to randomisation, whether before or after stroke/TIA. Data are number (%) or mean (standard deviation). Comparisons by one-way analysis of variance.

	All	Aspirin	Asp/Clop^†^	Asp/Dip	Clopidogrel	None	2*p*
Number (%)	685 (22.1)	426 (20.9)	42 (25.6)	50 (23.3)	54 (34.2)	111 (21.6)	
Mean (SD)	93 (39)	91 (35)	88 (27)	104 (82)	90 (27)	98 (32)	0.069

## Additional Points

*Recruiting Sites.* Sites are number of patients with baseline and/or on-treatment P-selectin measurement. The following 23 UK-based TARDIS sites took part in the P-selectin substudy: Ayr (2), Bath (14), Bury (4), Chester (12), Chesterfield (11), Coventry (11), Derby (33), Durham (5), Exeter (11), Glasgow Royal (9), Guildford (10), Ipswich (39), London Croydon (1), London King's College (103), London Princess Royal (13), London St Bartholomew's (24), London St George's (164), Mansfield (47), Nottingham (176), Preston (4), Stoke-on-Trent (46), Upton (3), and Wolverhampton (7).
